# ClassTR: Classifying Within-Host Heterogeneity Based on Tandem Repeats with Application to *Mycobacterium tuberculosis* Infections

**DOI:** 10.1371/journal.pcbi.1004475

**Published:** 2016-02-01

**Authors:** Leonid Chindelevitch, Caroline Colijn, Prashini Moodley, Douglas Wilson, Ted Cohen

**Affiliations:** 1 Department of Epidemiology of Microbial Diseases, Yale School of Public Health, New Haven, Connecticut, United States of America; 2 Department of Mathematics, Imperial College, London, United Kingdom; 3 School of Laboratory Medicine and Medical Sciences, Nelson R Mandela School of Medicine, University of KwaZulu-Natal, Durban, South Africa; 4 Department of Medicine, Edendale Hospital, Pietermaritzberg, South Africa; 5 Nelson R Mandela School of Medicine, University of KwaZulu-Natal, Durban, South Africa; University of New South Wales, AUSTRALIA

## Abstract

Genomic tools have revealed genetically diverse pathogens within some hosts. Within-host pathogen diversity, which we refer to as “complex infection”, is increasingly recognized as a determinant of treatment outcome for infections like tuberculosis. Complex infection arises through two mechanisms: within-host mutation (which results in clonal heterogeneity) and reinfection (which results in mixed infections). Estimates of the frequency of within-host mutation and reinfection in populations are critical for understanding the natural history of disease. These estimates influence projections of disease trends and effects of interventions. The genotyping technique MLVA (multiple loci variable-number tandem repeats analysis) can identify complex infections, but the current method to distinguish clonal heterogeneity from mixed infections is based on a rather simple rule. Here we describe ClassTR, a method which leverages MLVA information from isolates collected in a population to distinguish mixed infections from clonal heterogeneity. We formulate the resolution of complex infections into their constituent strains as an optimization problem, and show its NP-completeness. We solve it efficiently by using mixed integer linear programming and graph decomposition. Once the complex infections are resolved into their constituent strains, ClassTR probabilistically classifies isolates as clonally heterogeneous or mixed by using a model of tandem repeat evolution. We first compare ClassTR with the standard rule-based classification on 100 simulated datasets. ClassTR outperforms the standard method, improving classification accuracy from 48% to 80%. We then apply ClassTR to a sample of 436 strains collected from tuberculosis patients in a South African community, of which 92 had complex infections. We find that ClassTR assigns an alternate classification to 18 of the 92 complex infections, suggesting important differences in practice. By explicitly modeling tandem repeat evolution, ClassTR helps to improve our understanding of the mechanisms driving within-host diversity of pathogens like *Mycobacterium tuberculosis*.

## Author Summary

Within-host heterogeneity of an infection can arise through two distinct mechanisms: within-host mutation and reinfection. While current genotyping techniques based on MLVA (multiple loci variable-number tandem repeat analysis) can identify within-host diversity, standard methods for classifying the mechanism driving this diversity have limitations. We present ClassTR, a novel approach for classifying these types of complex infections. ClassTR uses optimization to resolve complex strains into simple strains and explicit models of tandem repeat evolution to classify the infections as clonal (due to within-host diversification) or mixed (due to reinfection). We illustrate ClassTR and validate its findings in the context of *Mycobacterium tuberculosis* infections. We construct simulated datasets to identify the best-performing variant of our method and find that it is significantly more accurate than the standard method of classification. We apply ClassTR to data from a study in South Africa and find substantial differences in the classifications produced by ClassTR and the standard method, demonstrating the real-world relevance of this approach. Our work suggests that an analysis of complex infections based on an evolutionary model improves our understanding of the drivers of within-host diversity.

## Introduction

The genotyping technique known as MLVA (multiple loci variable-number tandem repeats analysis), which identifies the number of copies of tandem repeat regions at specific pre-selected loci, has benefited the study of many bacteria. Data produced by MLVA can be used to glean information about bacterial lineage, pathogenicity and relation to other bacteria of the same species [1]. Our study focuses on a specific bacterium, *Mycobacterium tuberculosis*, but our methods are generally applicable to a variety of bacteria.

Genetic and genomic approaches for interrogating the composition of *Mycobacterium tuberculosis* infections occurring within individuals has in some settings revealed an impressive degree of complexity, reflecting both within-host mutation and reinfection as distinct routes to complexity [2]. These complex infections, especially those comprising both drug-susceptible and drug-resistant isolates (i.e. heteroresistance), can undermine the effective treatment of individual patients [3–5], complicate laboratory testing and evaluation of treatment programs [2], and affect the transmission dynamics of disease in communities [6, 7]. While an individual’s clinical response to treatment may not depend on whether heteroresistance has arisen through within-host mutation or by reinfection, our ability to distinguish these mechanisms has profound implications for our understanding of the natural history of disease and for projections of disease trajectories. For example, high contributions of reinfection indicate limited immune protection associated with previous infection, and have implications for the impact of new and existing vaccines [8] and for the effectiveness of preventive therapy [9]. High contributions of within-host mutation would affect expected rates of acquired resistance and would have implications for optimal antibiotic dosing strategies [10, 11]. Accordingly, accurate estimates of the prevalence of complex infections among tuberculosis patients and new methods for distinguishing the relative contributions of within-host mutation and reinfection to within-host diversity would be valuable.

**Fig 1:**
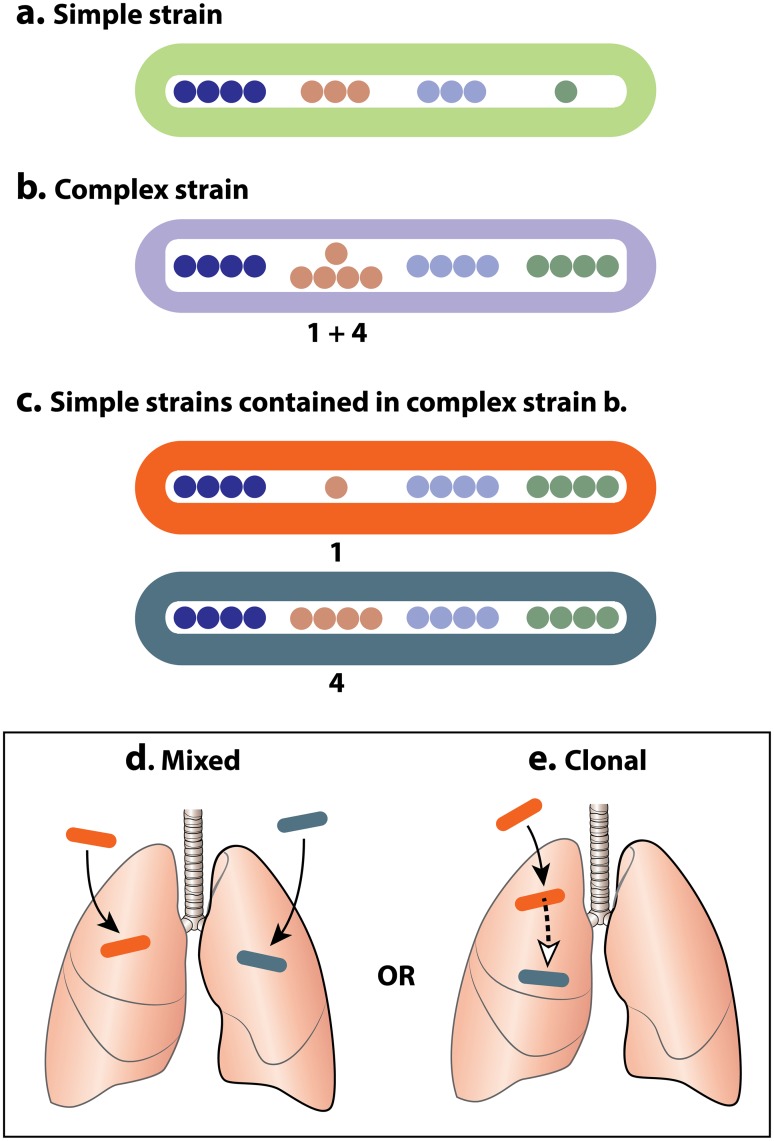
MIRU-VNTR and complex infections. **a**. A simple strain **b**. A complex strain **c**. The two strains contained in the complex strain; they can result from either **d**. a clonally heterogeneous infection or **e**. a mixed infection.

Mycobacterial interspersed repetitive unit-variable number tandem repeat (MIRU-VNTR), the specific name of the MLVA technique for *Mycobacterium tuberculosis*, is a currently favored approach for genotyping strains and offers advantages for detecting within-host heterogeneity over other methods such as spacer oligonucleotide sequencing (spoligotyping) and restriction fragment length polymorphism analysis (RFLP). These molecular genetic approaches for TB genotyping are reviewed in Mathema et al. [12] and an evaluation of their utility for detecting complex infections is described by Cohen et al [2]. MIRU-VNTR is a microsatellite typing system which produces a readout containing the number of copies of a repeat region at several pre-selected loci [13, 14]. These copy number variants (CNVs) can then be used to compare the *Mycobacterium tuberculosis* strain to other similarly typed strains. If a patient harbors a complex infection, there will frequently be 2 (and sometimes 3) different CNVs at a single locus, and these can result from a clonally heterogeneous or a mixed infection. This situation is illustrated in Fig 1.

Classifying complex MIRU-VNTR patterns as being due to either within-host mutation or reinfection is challenging. The current accepted approach for distinguishing clonal heterogeneity from mixed infection is a simple rule-based method: if two or more loci have multiple CNVs the infection is classified as “mixed”, whereas if only one locus has multiple CNVs the infection is classified as “clonally heterogeneous” [13–15]. This approach is sensible given that the more complexity observed within a particular genotype, the more likely it is to be due to reinfection with a distinct second strain. In addition, there are several sources of evidence which suggest that clonal evolution occurring over a relatively short period is unlikely to result in multiple complex loci [16–18]. Nonetheless, the rule this approach is based on suffers from several limitations. First, it does not take the context of the infection into account (namely, whether the constituent strains are present in other members of the population). Second, it does not distinguish between copy numbers that are a small genetic distance apart (such as 3 and 4) from ones that are far apart (such as 3 and 15), even though clonal heterogeneity is less plausible in the latter case. Third, this approach does not facilitate the resolution of mixed infections into their constituent strains.

We propose a new method, which we call ClassTR, to classify complex infections using MLVA data. Our method is based on an established model of tandem repeat evolution that accounts for the stepwise character of mutations, which we extend by using differential rates of evolution for different loci. ClassTR leverages the entire set of isolate genotypes collected in a population in order to resolve complex strains into simple strains (i.e. strains with only one CNV at each locus). Then, using a model of tandem repeat evolution it identifies the most likely sources of each simple strain to establish the probability of each patient having a mixed infection. We show that ClassTR outperforms the standard rule-based method, reducing its error rate by 61% on simulated data, and produces significantly different classifications than the standard method on a dataset collected from a community in KwaZulu-Natal, South Africa. ClassTR is implemented in the R Statistical Computing Language [19] in **Supplementary Materials** (S1 Code).

## Results

### The ClassTR algorithm for classifying complex infections

**Fig 2:**
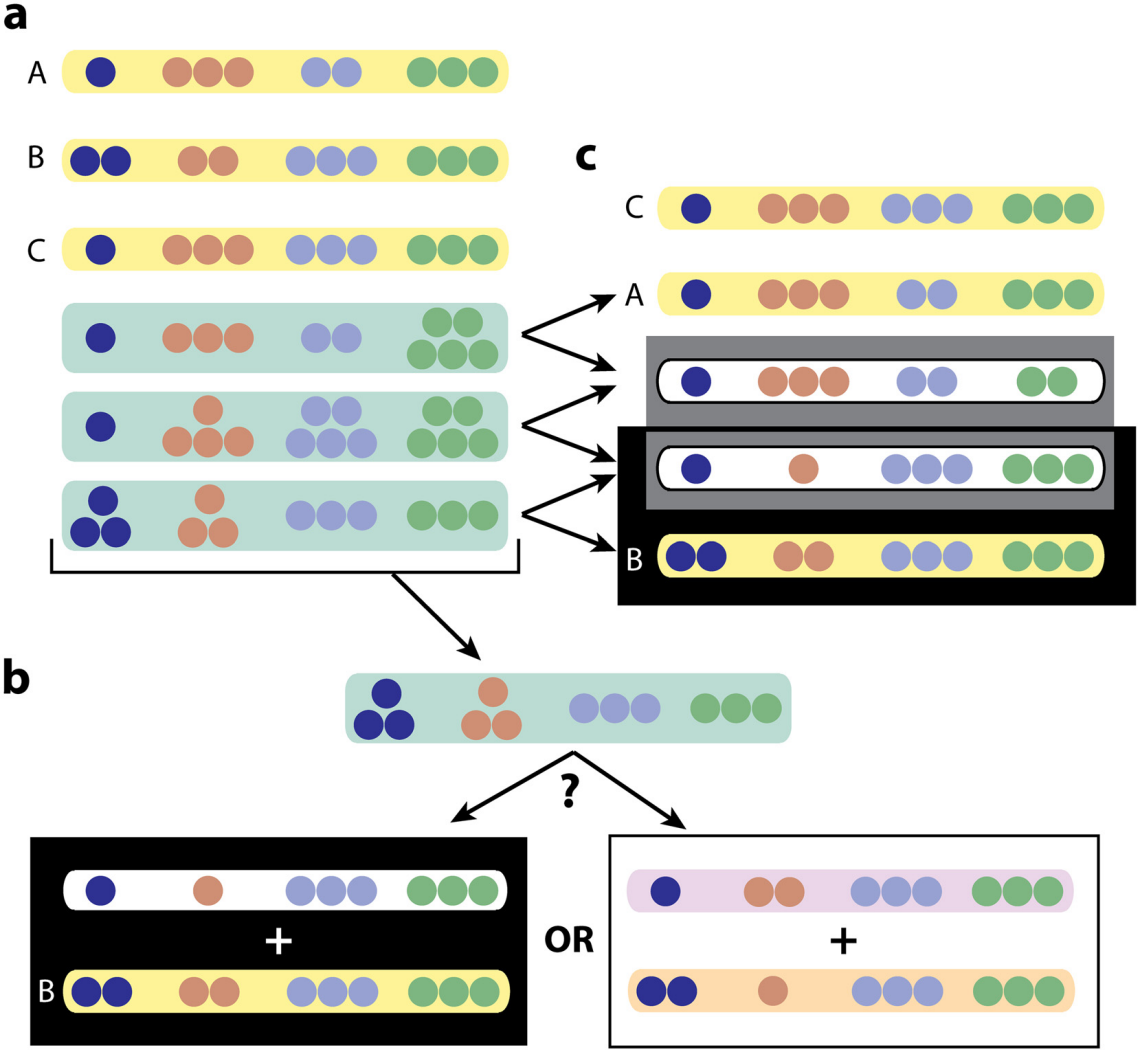
Example of the ClassTR resolution. **a**. A dataset containing 3 simple strains (*F* in the problem description) and 3 complex strains (*C* in the problem description) **b**. The 2 possible resolutions of the third complex strain (*q* = 2) into simple strains (the first complex strain has 1 possible resolution (*q* = 1) and the second one, 4 possible resolutions (*q* = 3)); the resolution chosen by ClassTR is framed **c**. The three initial (*F* in the problem description) and two newly added (*S* in the problem description) simple strains that form the unique optimal solution, with arrows indicating the resolution of complex strains into simple strains

**Fig 3:**
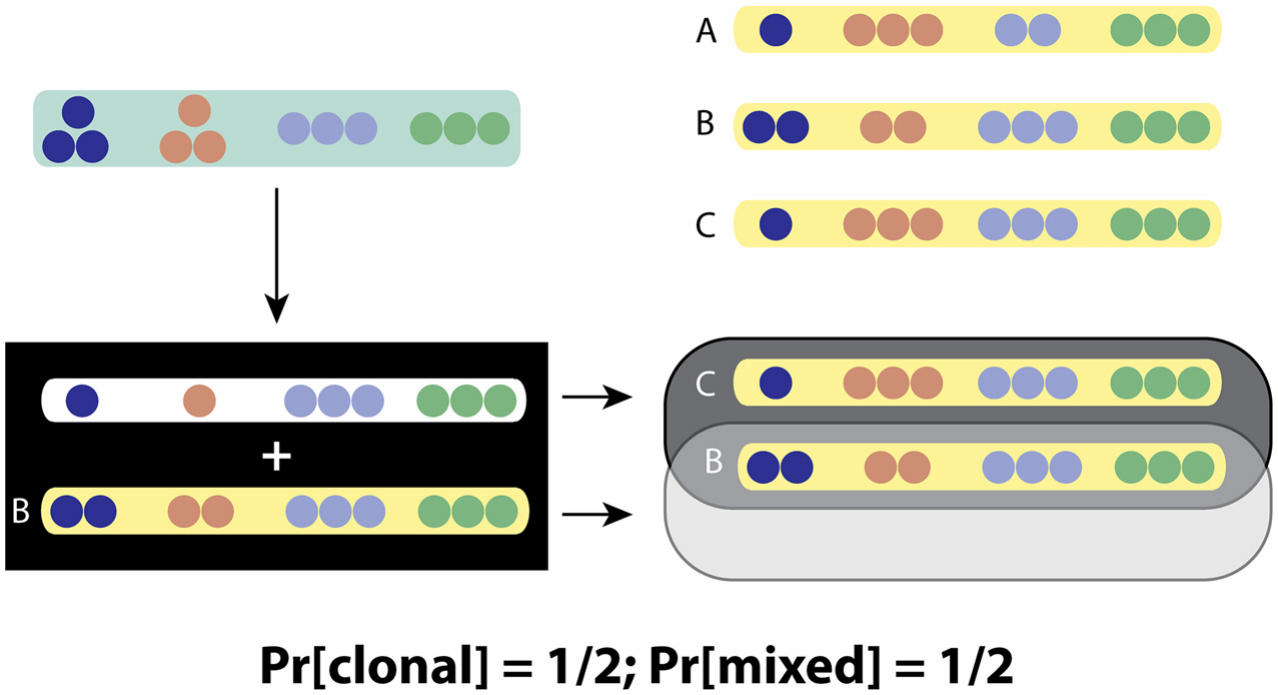
Example of the ClassTR classification. **Top right** The precursor sets (in the unweighted constant metric) indicated by arrows from the simple strains chosen to cover the third complex strain **Bottom** Its final classification given by ClassTR

We say that a patient harbors a *complex infection* if at least one of the MIRU-VNTR loci contains 2 different CNVs. We assume that there are always 1 or 2 CNV per locus, and this is indeed what we usually observe in practice. The ClassTR algorithm classifies these complex infections as resulting from either clonal heterogeneity or mixed infection (when both clonal heterogeneity and mixed infection are present, ClassTR classifies the infection as mixed). It includes three steps, each of which is briefly discussed below and described in more detail in the **Methods** section. Briefly, ClassTR starts by creating an optimization problem to resolve the complex strains into their constituent simple strains. After solving this optimization problem, ClassTR uses the resulting simple strain representation of complex strains to infer the possible provenance of each of these complex strains. Finally, it computes the probability of clonal heterogeneity for each patient with a complex infection. We illustrate this process on a small example with 3 simple and 3 complex strains in Figs 2 and 3.

### Defining a distance between strains

Following Aandahl et al [20] we define distances between strains based on explicit models of tandem repeat evolution: a constant model and a linear model. Both models assume that copy numbers evolve in a stepwise fashion, consistent with the process of slipped-strand mispairing [21]. The constant model assumes a Poisson process at each locus by which the copy number increases or decreases by 1 at a constant rate, while the linear model assumes a Poisson process at each copy, so that the rate of mutation is proportional to the current copy number. In both cases the distance between two strains represents the total number of mutation events required to go from one to the other. In addition to these basic models where different loci undergo mutations at the same rate, we also consider weighted models in which different loci mutate at different rates. In order to estimate these locus-specific mutation rates we use measures of locus diversity.

### Covering complex strains with simple ones

We say that a set of simple strains *exactly covers* a complex strain if the set of CNVs at each locus of the simple strains is precisely the CNVs at the corresponding locus of the complex strain. In general, a strain with one or more complex loci having 2 CNVs each can be exactly covered by 2 simple strains. However, in the absence of additional constraints there can be as many as 2^*q*−1^ possible such covers of a strain with *q* complex loci, which is 2048 possibilities for *q* = 12, the largest we observe in our data. We make a parsimony assumption and search for the covers of the complex strains that introduce the smallest possible number of additional simple strains (i.e. ones not observed in the original dataset). This defines an optimization problem which may have multiple solutions, especially when the cases are not densely sampled from the population. We narrow down alternative possibilities by a system of rewards for using a strain frequently observed in the dataset and penalties for strains that are far removed from any other simple strains in the dataset.

### Computing predecessors and the final classification

Once the optimization problem is solved, every complex strain is represented as a superposition of simple strains. These simple strains can be present in the original dataset or newly added. For each of the newly added simple strains we compute one or more predecessors among the original simple strains, defined as the closest among these strains according to the distance we chose. The final probabilities are then obtained by comparing the sets of predecessors of the two strains constituting a given complex strain; the more similar they are, the more likely the strain is to be the result of clonal heterogeneity.

### Classification of complex infections in a South African dataset

The South African dataset we work with consists of data collected during a prospective study of within-host diversity of *M. tuberculosis*. Briefly, 500 adult, sputum smear-positive TB patients in a geographic cluster of participating clinics in KwaZulu Natal were sequentially recruited for participation at the time of diagnosis and before treatment was initiated. Additional pre-treatment sputum was collected from each participant and cultured in solid and liquid media. Bacterial DNA was isolated from both media and genotyping was done by 24 loci MIRU-VNTR according to standardized protocols [14]. Out of the 500 study participants, the isolates of 436 (87%) were successfully typed and included in this study. Of the 436 patients included in the study, 92 (21%) had complex MIRU-VNTR patterns. We note that, like many other South African communities, the one in this study has a high HIV prevalence.

The standard rule-based classification method designated 44 of 92 of the complex strains as clonal and the remaining 48 as mixed, whereas our method classified 50 of them as clonal and 42 as mixed. Only one patient got assigned a probability of 1/4 for clonality and 3/4 for mixed, and we ended up using the majority rule and classifying their infection as mixed. There were a total of 18 discrepant calls, 6 in which a strain was called clonal by the standard method but mixed by ours, and 12 in which the reverse occurred. Our results are summarized in Table 1.

**Table 1:** Classification results of the standard method and ClassTR on the South African dataset.

Standard/ClassTR	Simple	Mixed	Clonal
Simple	344	0	0
Mixed	0	36	12
Clonal	0	6	38

### Validation of the method on simulated data

In order to evaluate the performance of the 8 different variants of our method (defined by the constant or the linear metric, as well as the commonly used Hamming metric and Goldstein metric, and weighted or unweighted loci), as well as the standard rule-based method based on the count of complex loci, we produced 100 simulated datasets with characteristics similar to our South African dataset. The details of our simulations are described in the **Supplementary Materials** (S1 Text). We attempted to match the original dataset in terms of its strain clustering characteristics, distribution of the number of complex loci in strains, and distribution of the differences between the CNVs in a complex locus. To this end, we simulated the evolution of an initial population of strains with random but constrained mutation and reinfection events a large number of times, selected a number of subpopulations of appropriate size, and selected the final datasets according to their distance to the two target distributions.

We selected 100 datasets of *N* = 415 strains each, *n* = 83(20%) of which were complex; 42 were the result of clonal heterogeneity and 41 were mixed infections. We applied the standard rule-based method and our method on each dataset. We evaluated the accuracy of each method as the average probability they assigned to the correct classification for the 83 complex strains; namely, if a method returned a probability *p* of clonality, we scored *p* if the complex strain was actually clonal and 1 − *p* if it was actually mixed. The accuracy of the standard rule-based method averaged 48%, not significantly different from the 50% that would be expected from a random classification. On the other hand, the accuracy of ClassTR using our metric of choice, the linear weighted metric, was 80%, for a 61% reduction in error. We also evaluated the correctness of the resolution of complex strains into their constituent simple ones, and found that ClassTR produced the correct resolution in 88% on clonal infections and 95% on mixed infections. The results of running different variants of our method on classification accuracy are shown in Table 2.

**Table 2:** Percent accuracy of classification methods on simulated datasets.

Metric	Hamming metric		Constant metric		Linear metric		Goldstein metric		Standard method	
Correct class	clonal	mixed	clonal	mixed	clonal	mixed	clonal	mixed	clonal	mixed
Unweighted	87%	68%	91%	65%	89%	66%	85%	64%	45%	52%
Weighted	87%	74%	90%	69%	89%	71%	85%	68%		

In addition, we created 9 groups of 100 datasets each, with similar characteristics but not constrained to resemble the original dataset as closely. Each group corresponded to a combination of one of three mutation rates (low, medium and high) and one of three reinfection rates (low, medium and high). Our method outperformed the standard rule-based method on all of them except for the classification at the low mutation rate. The results of running ClassTR on those datasets are shown in Table 3 (for the resolution, using randomly picked resolutions as the baseline) and Table 4 (for the classification, using the standard method as the baseline). They suggest that although the resolution performance of ClassTR deteriorates quite substantially on mixed infections at high mutation rates, its classification performance remains consistently good, both in absolute terms and in comparison with the standard method. This finding is further substantiated by Table 5, which shows that ClassTR finds the correct classification when the correct resolution is given most of the time, with a slight deterioration at high mutation rates. However, it is also able to find the correct classification from an incorrect resolution quite frequently, as evidenced by a comparison of all three tables.

**Table 3:** Percent accuracy of ClassTR’s strain resolution on 9 simulated datasets; mutation rates: low, 0.00012; medium, 0.0012; high, 0.012; reinfection rates: low, 0.012; medium, 0.04; high, 0.12 (per year); the percentage in brackets is the corresponding accuracy for the method of picking a random resolution.

Mutation/Reinfection	Low mutation		Medium mutation		High mutation	
Correct class	clonal	mixed	clonal	mixed	clonal	mixed
Low reinfection	100% (98%)	87% (3.8%)	97% (93%)	79% (5.3%)	82% (53%)	32% (2.7%)
Medium reinfection	100% (99%)	87% (3.9%)	98% (93%)	80% (5.8%)	81% (54%)	32% (2.9%)
High reinfection	100% (99%)	88% (3.2%)	97% (93%)	80% (5.5%)	78% (54%)	33% (2.2%)

**Table 4:** Percent accuracy of ClassTR’s classification on 9 simulated datasets; mutation rates: low, 0.00012; medium, 0.0012; high, 0.012; reinfection rates: low, 0.012; medium, 0.04; high, 0.12 (per year); the percentage in brackets is the corresponding accuracy for the standard method.

Mutation/Reinfection	Low mutation		Medium mutation		High mutation	
Correct class	clonal	mixed	clonal	mixed	clonal	mixed
Low reinfection	96% (96%)	99% (99%)	97% (86%)	96% (98%)	88% (33%)	95% (99%)
Medium reinfection	96% (97%)	99% (99%)	94% (86%)	96% (97%)	82% (34%)	96% (99%)
High reinfection	94% (98%)	99% (99%)	92% (87%)	97% (97%)	74% (34%)	97% (99%)

**Table 5:** Accuracy of ClassTR’s classification on 9 simulated datasets conditional on correct resolution.

Mutation/Reinfection	Low mutation		Medium mutation		High mutation	
Correct class	clonal	mixed	clonal	mixed	clonal	mixed
Low reinfection	96%	99%	97%	96%	89%	94%
Medium reinfection	96%	99%	94%	96%	84%	95%
High reinfection	94%	99%	92%	97%	76%	97%

## Discussion

ClassTR is the first alternative method for classifying complex bacterial strains as either clonally heterogeneous or mixed infections. In contrast with the existing rule-based classification method, it includes an explicit model of tandem repeat evolution and utilizes information from other strains collected locally, provides probabilistic rather than deterministic classifications, and allows for the identification of individual strains within complex infections.

There are two computational problems that ClassTR solves. The first one, which we called the Parsimonious Resolution Problem and showed to be NP-complete, is reminiscent of haplotyping problems in eukaryotic genomes [22]. The key difference is the haploid nature of the bacterial genomes we analyze; the observed complexity in the strains is the result of the genotyping technique we use rather than an actual allelic variation due to recombination. The second one, which is the classification problem for complex bacterial strains, is reminiscent of the type of problems that arise in cancer genomics [23] in deciding whether particular tumor genotypes are related to one another (similar to clonal heterogeneity) or have arisen independently (similar to mixed infection). The key difference is the evolutionary model we adopt for tandem repeats, which may not apply to cancer.

At the current time we lack a “gold standard” approach for determining which infections are actually complex, and whether these complex infections are due to within-host mutation or reinfection. This makes it challenging to evaluate the relative performance of the standard rule-based approach and ClassTR. While future advances in genome sequencing are likely to provide additional data to test ClassTR against the standard rule-based approach, our results on simulated data suggest that ClassTR provides more accurate classifications than the standard approach, and the additional computational resources are justified by the improvement in classification accuracy. While the performance of ClassTR and the standard method for classification of complex infections is similar if the true mutation rate of MIRU-VNTR loci is at the lowest end of the plausible range, ClassTR outperforms the standard method under scenarios with higher mutation rates within this range. Furthermore, applying ClassTR to our data from KwaZulu Natal generates results that are substantially different from the standard rule-based method, which demonstrates that the difference between these approaches is not just theoretical. In addition, ClassTR provides the only available approach to extract the constituent strains involved in a mixed infection from MIRU-VNTR data alone.

There are several opportunities to modify the models we have used to even better reflect the evolutionary process driving copy number variation. First, our evolutionary model does not account for the fact that some evolutionary events may duplicate multiple segments in a single timestep. A more sophisticated model might allow for such duplication events to happen, albeit at a small rate, and this rate could be estimated from available data. Second, we model copy number increases and decreases symmetrically, whereas a more flexible model could allow these events to occur at different rates. Finally, an alternate model might be needed to account for the possibility that two strains may be simultaneously transmitted from one person to another or for the possibility of having more than two strains within a host, which may be relevant for certain types of infectious pathogens [24]. In addition, an intriguing opportunity for future work would be to investigate how accurate the classifications of complex infections as clonal or mixed could be at the time that each patient is admitted, rather than at the end of the study as we have done here, as well as to take into account the information about the strains found in a patient’s contacts, such as household contacts, perhaps by using these to constrain resolutions.

In conclusion, ClassTR is a tool which we believe will advance our capacity to identify the mechanisms underlying within-host heterogeneity in TB and other bacteria. By distinguishing within-host mutation from reinfection, we anticipate that this method will improve our understanding of the natural history of pathogenic infection at the individual patient level, and will improve our ability to project transmission dynamics and the effects of interventions in communities.

## Methods

This part of the paper is organized as follows. First, we define simple and complex strains, and explain how simple strains can cover complex strains. These definitions allow us to formulate our first problem, that of resolving the complex strains into simple strains by introducing as few simple strains unobserved in the data as possible. In the **Supplementary Materials** (S1 Text) we show that this problem is NP-complete. We continue by showing how this problem can be efficiently solved using a mixed integer linear programming formulation. In the **Supplementary Materials** (S1 Text) we also explain how this problem can be simplified using graph decomposition, and how the number of solutions can be further reduced by using an idea from information theory known as the *minimum description length*. We conclude by describing the model of tandem repeat evolution and the method we use to classify complex strains as arising from clonal heterogeneity or mixed infection. In the **Supplementary Materials** (S1 Text) we further elaborate on some alternative models we have considered and how they influence our results.

### Covers of complex strains

In this section we define simple and complex strains and describe the principled way in which ClassTR separates complex strains into simple strains.

We formally define a *simple strain* as a string of length *L* (for MIRU-VNTR, *L* = 12 or *L* = 24) over the alphabet *A* consisting of all integers from 0 to some upper bound *t*_max_. If **s** is a simple strain, we denote by **s**_*j*_ its *j*-th symbol. We define a *complex strain* as a string of length *L* over the alphabet P(A), the power set of *A*, so that each of its symbols is a subset of *A*. If **s** is a complex strain, we call **s**_*j*_ the *content* of **s** at position *j*.

A collection *C* of simple strains will be called a *cover* for a complex strain **s** if at each position 1 ≤ *j* ≤ *L*, we have **s**_*j*_ ⊂ ∪_**c**∈*C*_ **c**_*j*_. In other words, the content of the strain **s** at each locus is included in *C*. A collection *C* will be called an *exact cover* for a complex strain **s** if equality holds, i.e. **s**_*j*_ = ∪_**c**∈*C*_ **c**_*j*_ ∀1 ≤ *j* ≤ *L*; in this case, *C* includes the content of **s** at each locus, and nothing else. A collection *C* will be called a *minimal (exact) cover* of **s** if *C* is an (exact) cover of **s** and no proper subset of *C* is. We always look for minimal exact covers for reasons of parsimony.

When a complex strain **s** has all contents of size 1 or 2, there exist minimal exact covers of size 2, and the number of such covers is 2^*q*−1^, where *q* is the number of positions with content of size 2. The value of *q* attains a maximum of 12 in our dataset, meaning that a single complex strain can have up to 2048 different covers.

Given the multiplicity of possible minimal covers for each complex strain, we use a global parsimony assumption to identify the ones that are actually present. Namely, we assume that, all other things being equal, the fewer simple strains we add to the ones in the dataset to cover all the complex strains, the better. Intuitively, this means that we attempt to explain complex infections in terms of strains we have observed as simple infections in the population. Thus we seek to cover all the complex strains by adding the smallest possible number of strains. Fig 2 presents a toy example of a dataset with its solution.

### The parsimonious resolution problem

In this section we formalize the problem of resolving the complex strains by introducing as few new simple strains as possible, which we call the parsimonious resolution problem. In the **Supplementary Materials** (S1 Text) we show that the decision version of this problem is NP-complete, even in the case of all copy number variants being 0 or 1. As a corollary, our proof establishes that the parsimonious resolution problem for spoligotype data (where a 0 indicates the absence and a 1 the presence of a particular region), a version of which was studied by Lazzarini et al [25], is also NP-complete.

The decision version of the parsimonious resolution problem (PRP) can be stated as follows.

Given: an integer *L*; a finite alphabet *A*; a set of strings *S* = {*s*_1_, *s*_2_, …, *s*_*k*_} of length *L* over *A* ∪ *A*^2^, but not entirely over *A* (i.e. each position contains 1 or 2 elements of *A*, with at least one position containing 2 elements of *A*); a set of “free” strings *F* = {*f*_1_, …, *f*_*m*_} of length *L* over *A*; an integer *K*.

Decide: whether there exists a collection *C* = {*c*_1_, *c*_2_, …, *c*_*K*_} of *K* strings of length *L* over *A*, such that, for each string *s* ∈ *S* there exist 2 strings *c* and *c*′ in *C* ∪ *F*, such that *c* ∪ *c*′ = *s* (where the union is taken component-wise).

The correspondence between the PRP and the problem we are actually solving is as follows: *L* is the number of loci, *A* is the set of possible CNVs, *S* are the complex infections present in the data and *F* are the simple infections present in the data. Finally, *K* is the number of additional (new) simple strains we are seeking to add to *F* in order to resolve all the complex infections.

### Integer linear program formulation

In this section we formulate the 0–1 integer linear program [26] for the parsimonious resolution problem. This integer linear program finds a set of simple strains that cover the complex strains in the dataset, paying for each simple strain that is not present in the dataset. It minimizes the total cost of these newly added simple strains. Its inputs are a set of simple strains that can be used “for free” and the set of complex strains to be covered. Its outputs are the variables corresponding to the new simple strains used in covering the complex strains.

Let *N* be the number of simple strains, *n* be the number of complex strains, and *q*_*i*_ be the number of complex loci in the *i*th complex strain. For simplicity we assume that there are exactly 2 copy number variants at each complex locus, which is the case for our dataset. Let *S*_*i*_ be the set of all simple strains that may be used to cover the *i*th complex strain, so that |*S_i_*| = *Q_i_* = 2^*q_i_*^. Let us also define $Q≔\sum_{i=1}^{n}Q_{i}$. Let $S≔\cup_{i=1}^{n}S_{i}$ and *q* := |*S*|. Note that *q* ≤ *Q*.

We define two categories of variables, one to indicate usage, and the other to indicate coverage. The usage variables are denoted *u*_*j*_ and are defined for every strain *j* in *S*. The value of *u*_*j*_ is 1 if the simple strain *j* is used in the cover of at least one complex strain, and 0 otherwise. The coverage variables are denoted *c*_*ij*_ and are defined for every complex strain *i* and every simple strain *j* in *S*_*i*_. The value of *c*_*ij*_ is 1 if the simple strain *j* is used to cover the complex strain *i*, and 0 otherwise. For a complex strain *i* and a simple strain *j* in *S*_*i*_, we denote by *i*∖*j* the *complement* of *j* in *i*, namely, the simple strain that, together with *j*, covers *i* (here we use the assumption that every complex locus has exactly 2 CNVs). The complement *i*∖*j* can be obtained by taking the CNV in each complex locus of *i* that was not used in *j*.

For each simple strain *j* in *S* we also define the cost *w*_*j*_ of adding it to the cover. The objective function is simply a linear combination of the usage variables *u*_*j*_ with the costs *w*_*j*_ as coefficients. We always take *w*_*j*_ = 0 if the simple strain *j* is present in the dataset, because it is already available to be used in a cover. We also take *w*_*j*_ = 1 for any simple strain *j* not present in the dataset, so the total cost ends up being the number of new strains used. The optimal solution is the one minimizing this total cost.

This leads us to the following integer linear program formulation:

$$\text{Minimize}\;\sum_{j}w_{j}u_{j}\;\text{subject}\;\text{to}$$ (1)

$$u_{j}\in \left\{0,1\right\}\;\forall \;j$$ (2)

$$c_{ij}\in \left\{0,1\right\}\;\forall \;i,j$$ (3)

$$c_{ij}=c_{i(i\backslash j)}\;\forall \;i,j$$ (4)

$$c_{ij}\leq u_{j}\;\forall \;i,j$$ (5)

$$u_{j}\leq \sum_{i}c_{ij}\;\forall \;j$$ (6)

$$1\leq \sum_{j}c_{ij}\;\forall \;i$$ (7)

The first two sets of constraints, Eqs (2) and (3), ensure that all the variables take values 0 or 1. The next set of constraints, Eq (4), ensures that the simple strain *j* is used to cover the complex strain *i* if and only if its complement simple strain, *i*∖*j*, is also used to cover the complex strain *i*. The next two sets of constraints ensure that the simple strain *j* is marked as used if (Eq (5)) and only if (Eq (6)) it is used to cover at least one complex strain *i*. Finally, the last set of constraints, Eq (7), ensure that the complex strain *i* is covered in at least one way by simple strains.

The number of *u*_*j*_ variables and constraints in Eq (2) is *q* ≤ *Q*; the number of *c*_*ij*_ variables and constraints in Eq (3) is *Q*; the number of constraints in Eq (4) is *Q*/2; the number of constraints in Eq (5) is *Q*; the number of constraints in Eq (6) is *q* ≤ *Q*; and the number of constraints in Eq (7) is *n*, for a total of *Q* + *q* ≤ 2*Q* variables and (5/2)*Q* + 2*q* + *n* ≤ (9/2)*Q* + *n* constraints. In particular, for our South African dataset, *n* = 92 and *Q* is roughly 8,000, while for our simulated datasets, *n* = 83 and *Q* varies from 5,000 to 25,000, so the total number of variables is always under 50,000 and the number of constraints under 100,000. Integer linear programs of this size can typically be solved to optimality in seconds by CPLEX (available from http://www-01.ibm.com/software/integration/optimization/cplex-optimizer). The total time required by ClassTR is about 5 minutes for the South African dataset with *N* = 436 strains. We additionally tested our method on a much larger dataset containing *N* = 4075 strains with *n* = 364 of them complex. Its processing took less than an hour on a single CPU, suggesting that our algorithm scales well with input size in practice.

### Distances on simple strains

In this section we define the four distances we use in ClassTR. These distances can be used to construct the predecessor sets which then allow us to calculate the probability of each complex strain being clonally heterogeneous or mixed.

We define the *constant metric* **d**_**C**_ between two simple strains as

$$d_{C}\left(s,s^{\prime }\right)=\sum_{j=1}^{L}\left|s_{j}-s_{j}^{\prime }\right|.$$

This corresponds to the minimum number of mutation events needed to get from one strain to the other in the constant model of tandem repeat evolution defined by Aandahl et al [20]. Indeed, since the constant model assumes a Poisson process at each locus, |*i* − *j*| is precisely the number of mutations required to get from *i* to *j*.

We also define the *linear metric* **d**_**L**_ between two simple strains as

$$d_{L}\left(s,s^{\prime }\right)=\sum_{j=1}^{L}\sum_{k=\min(s_{j},s_{j}^{\prime })+1}^{\max(s_{j},s_{j}^{\prime })}\frac{1}{k}.$$

This corresponds to the expected number of timesteps needed to get from one strain to the other in the linear model of tandem repeat evolution defined by Aandahl et al [20]. Indeed, since the linear model assumes that a Poisson process takes place at each copy, it takes an expected 1/*m* timesteps to go from *m* to *m* − 1 copies.

Two other standard metrics we use are the *Goldstein metric* **d**_**G**_ and the *Hamming (categorical) metric* **d**_**H**_, respectively defined as

$$d_{G}\left(s,s^{\prime }\right)=\frac{1}{L}\sum_{j=1}^{L}{\left(s_{j}-s_{j}^{\prime }\right)}^{2}\;\text{and}\;d_{H}\left(s,s^{\prime }\right)=\sum_{j=1}^{L}\left[s_{j}\neq s_{j}^{\prime }\right],$$

where [*I*] is the Iverson bracket whose value is 1 if expression *I* is true and 0 otherwise. Note that the Goldstein metric is not a metric in the traditional sense because it does not respect the triangle inequality.

In addition we define weighted analogs of all these metrics, which are obtained by multiplying the contribution of each locus by its weight. To estimate the weight of each locus ClassTR uses the Simpson index [27], also known as the Hunter-Gaston index [28], reasoning that the more diverse a locus is, the faster it evolves and the less weight it should carry. These weights then allow us to compute the corresponding weighted distances in the constant or linear models defined below, which we denote $d_{c}^{w}$ and $d_{L}^{w}$, respectively. In our datasets these weights ranged from 0.16 to 1.

### Predecessor sets and classification

Given the set of simple strains generated by the optimization, we describe how to produce the final soft classification of complex strains along the clonally heterogeneous to mixed spectrum in this section.

We start by choosing a distance function **d** on simple strains. Given a strain *j*, we define the *predecessor set* *P*(*j*) as the subset of the simple strains *S* present in the original dataset that are closest to *s* according to **d**. Formally,

$$P\left(j\right)≔\arg\min_{s\in S}d\left(j,s\right).$$

Of course, for any strain *s* ∈ *S*, the predecessor set only contains *s* itself (we do not take the presence of duplicate strains into account). We also note that the more highly resolving the distance, the smaller the predecessor sets are going to be. Thus, the unweighted constant distance could give rise to ties for the closest strain, but the weighted constant distance or the linear distance is less likely to yield a tie.

Intuitively, the more similar the predecessor sets of the constituent strains are to each other, the more likely the complex strain is to be clonally heterogeneous. For example, if two different covering strains are both very close to the same simple strain in the dataset, the complex strain composed of both of them is more likely to be clonal than if the two strains’ nearest matches in the data are two very different strains. We formalize this by using the Jaccard index [29] to evaluate the similarity of two sets *A* and *B*, defined as the size of their intersection divided by the size of their union:

$$J\left(A,B\right)≔\frac{\left|A\cap B\right|}{\left|A\cup B\right|}.$$

Suppose that *A* and *B* are the predecessor sets of the constituent strains of a complex strain. Then we take the Jaccard index of *A* and *B* as the probability of the complex strains being clonally heterogeneous. Thus, a complex strain covered by two strains with identical predecessor sets will be classified as clonally heterogeneous, while one with two strains with non-overlapping predecessor sets (for example one covered by two distinct simple strains present in the original dataset) will be classified as mixed, with intermediate variants also possible as shown in Fig 3. This probability is the value we report as our final classification.

## Supporting Information

S1 Text Additional details on the methods used: NP-completeness proof; minimum description length formalism; strain intersection graph; enumeration of optimal solutions; details of the simulation approach; and selection of the best method for ClassTR. (PDF)

S1 Code The R package implementing the ClassTR method. The zip file must be unzipped using a non-recursive decompression utility that preserves the internal file structure. The extracted package can then be installed via the R command *install.packages(pkgs = “ClassTR_0.1.0.tar.gz”, repos = NULL)* from the directory it is unzipped to.
(ZIP)
