# Correction: ClassTR: Classifying Within-Host Heterogeneity Based on Tandem Repeats with Application to *Mycobacterium tuberculosis * Infections

**DOI:** 10.1371/journal.pcbi.1004806

**Published:** 2016-02-29

**Authors:** The PLOS Computational Biology Staff

There is a typographical error in the legend for Figure 1.

The statement “they can result from either **d.** a clonally heterogeneous infection or **e.** a mixed infection” should instead read “they can result from either **d.** a mixed infection or **e.** a clonally heterogeneous infection”.
