# Supplementary material for: ClassTR: Classifying Within-Host Heterogeneity Based on Tandem Repeats with Application to Mycobacterium tuberculosis Infections
Source: PLoS Comput Biol. 2016 Feb 1;12(2):e1004475. doi: 10.1371/journal.pcbi.1004475 (PMC4734664; doi:10.1371/journal.pcbi.1004475)
Supplement: S1 Text — (PDF) [file pcbi.1004475.s001.pdf]

Supplementary Materials

The NP-completeness of the parsimonious resolution problem

The parsimonious resolution problem (henceforth abbreviated as PRP) is clearly in NP since a proposed collection C, together with a certificate listing pairs of elements of $C \cup F$ that create the elements of S, can be checked to be a correct solution in time linear in k, L and m.

For the NP-completeness proof, we will consider the special case $A = \{0, 1\}$ and $F = \{0^L\}$ (i.e. the only free string is the all-zero string). We will further restrict the problem so that every position of every string in S is either 0 or $\{0, 1\}$. We will show that this special case is equivalent to a problem known to be NP-complete.

We start by noting that in any decomposition of a string s_j as $c \cup c'$, both c and c' need to have a 0 in each position where s_j has a 0, and exactly one of them needs to have a 1 in each position where s_j has a $\{0, 1\}$. If $f_1 = 0^L$ is used in a decomposition of s_j, then the complement c will have a 1 wherever s_j has a $\{0, 1\}$. Let $B = \{1, 2, ..., L\}$. Then, by identifying the string s_j with the subset B_j of positions that have a $\{0, 1\}$, and similarly identifying the string c_i with the subset C_i of positions that have a 1, the problem becomes equivalent to the following one:

Given: a finite ground set B and a collection of subsets $\{B_1, B_2, ..., B_k\}$; an integer K.

Decide: whether there exists a collection $C = \{C_1, C_2, ..., C_K\}$ of K subsets of B, such that every B_j is either in the collection C or can be written as the disjoint union of exactly two members of C: $B_j = C_k \biguplus C_l$.

However, this special case of the PRP is precisely the restricted version of the normal set basis problem (NSBP) where each set in the collection is restricted to be covered by at most two subsets, which was proved to be NP-complete by Jiang and Ravikumar [1]. More precisely, since all the decompositions in Jiang and Ravikumar's reduction from Vertex Cover to NSBP have either a single set or two disjoint sets, this restriction of the NSBP is as hard as the general case, completing the proof.

Minimum description length

In most simulated and real datasets the number of possible optimal solutions is large, varying from 10^{15} to 10^{20}. This is because the number of possible resolutions of a complex strain in the data can go up to 2^{11}, and since a simple strain can occur in the resolution of multiple complex strains, the total number of possible solutions scales like 2^{11n}. For this reason we employ the minimum description length (MDL) formalism to derive a secondary optimization criterion, which typically reduces the number of optimal solutions to 10^5 to 10^{10}. The MDL formalism was introduced by Rissanen [2] to extend the parsimony principles behind Occam's razor to situations where a choice must be made between many competing hypotheses of varying complexity. It has previously been used to solve a variety of computational biology problems involving the identification of disease-associated SNPs [3], infection progression markers [4], and the inference of gene regulatory networks [5]. In our setting, we use MDL in the following way. Each cover of complex strains with simple strains using the minimal number of new simple strains is a hypothesis. The encoding of the hypothesis contains three pieces: (a) the simple strains in the original dataset; (b) the newly added strains; (c) for each complex strain, two pointers to the simple strains that cover it.

We will now define a specific encoding (or description) that we will use. This encoding will favor the use of simple strains frequently found in the data, and penalize newly added simple strains that are too distant from the simple strains in the dataset. The winning hypothesis will be the one that has the shortest encoding among all competing hypotheses. We will encode each of (a), (b) and (c) separately, but since only the latter two will differ between resolutions, we will focus exclusively on them in the following description.

We will treat the new strains (part b) as the irregular part of the data. To encode a newly added simple strain j we will proceed as follows. First, find a strain s in the data that is closest to j. Second, enumerate the k loci $1 \leq l_1 \leq \cdots \leq l_k \leq 24$ at which they differ, where $k := \mathbf{d_H}(j, s)$. As is standard in MDL, we will use the binary system, so this part of the encoding will require $k \cdot \log_2 24$ bits. Second, for each locus l_i we will encode the direction of change (up or down) that we need to take in order to obtain the value of j_{l_i} from the value of s_{l_i}. This will take an additional bit per locus (we can define 0 to mean up and 1 to mean down), for an extra k bits. Finally, for each locus l_i we will encode the magnitude of change, $|j_{l_i} - s_{l_i}|$. Since this will typically be small, we will use the unary encoding, so the total additional number of bits will be $\mathbf{d_C}(j, s)$. This gives a total of

$$w_j := \min_{s \in S} \big(\log_2(24)\mathbf{d_H}(j,s) + \mathbf{d_H}(j,s) + \mathbf{d_C}(j,s)\big) = \min_{s \in S} \big(\mathbf{d_C}(j,s) + (4 + \log_2 3)\mathbf{d_H}(j,s)\big),$$

where we take the minimum over all simple strains $s \in S$ in order to achieve the most efficient encoding. Note that we can directly use the w_j as weights in the integer linear programming formulation above to obtain the total cost of part (b).

To encode the covers (part c) we will now create pointers to the simple strains in parts (a) and (b). According to information-theoretic principles (implemented, for instance, in Huffman coding), the optimal length of a pointer to an element occurring with probability p is $-\log_2(p)$. We will use the empirical frequency $n_j/(N+K)$ for the simple strain j, where $N := |S|$ is the number of simple strains in the dataset and K is the number of newly added simple strains. Here, $n_j = 1$ for any newly added simple strain j, and $n_j \geq 1$ for simple strains j in the dataset. Note that the c_{ij} variables in the integer linear programming formulation allow us to conveniently represent the total length of these pointers as a sum over i and j. We see that their contribution is given by

$$\sum_{i,j} c_{ij} \left[-\log_2 \frac{n_j}{N+K}\right] = \sum_{i,j} c_{ij} \log_2(N+K) - \sum_{i,j} c_{ij} \log_2(n_j) = Z - \sum_{i,j} c_{ij} \log_2(n_j),$$

where $Z := 2n \log_2(N+K)$ is a constant independent of the resolution, and can therefore be ignored for the purposes of optimization (the minimality of K helps us ensure that we only need to consider the second term in the sum).

The final MDL encoding will then be the solution of the following optimization problem:

$$\begin{aligned}
\text{Minimize } & \sum_j w_j u_j - \sum_{i,j} c_{ij} \log_2(n_j) \text{ subject to} \\
u_j &\in \{0,1\} \;\forall\, j \\
c_{ij} &\in \{0,1\} \;\forall\, i,j \\
c_{ij} &= c_{i(i\backslash j)} \;\forall\, i,j \\
c_{ij} &\leq u_j \;\forall\, i,j \\
u_j &\leq \sum_i c_{ij} \;\forall\, j \\
1 &\leq \sum_j c_{ij} \;\forall\, i \\
K &= \sum_{j \in S} u_j.
\end{aligned}$$

Here, K is the optimal number of newly added simple strains and the w_j are defined by the "hybrid distance" function which is a linear combination of $\mathbf{d_H}$ and $\mathbf{d_C}$.

Strain intersection graph

In this section, we describe how the problem of optimally resolving a set of complex strains into simple ones can be simplified by using a graph decomposition procedure. This is necessary in order to render the number of solutions manageable.

Let us construct an undirected *strain intersection graph* $G = (V, E)$, where the vertices V correspond to each of the complex strains, and there is an edge from j to k if and only if complex strains j and j' contain a simple strain that can be used to cover both of them. Formally, the edge set is $E = \{(j, j')|j_k \cap j'_k \neq \emptyset \;\forall k, 1 \leq k \leq L\}$. Equivalently, there is an edge from j to j' if and only if there exist exact covers $\{i, j\backslash i\}$ for j and $\{i, j'\backslash i\}$ for j' for some simple strain i.

It is then easy to see (and we formally prove in the Lemma below) that the optimization problem can be solved separately for each connected component of G. Intuitively, the choices made for one of the connected components cannot influence the choices made for any other connected component.

Lemma Let $G = (V, E)$ be as above and let $c_1, \ldots, c_l$ be the connected components of G. Then any optimal solution of the optimization problems defined in the previous two sections is obtained by combining one optimal solution for each of the $c_k, 1 \leq k \leq l$, and every optimal solution can be decomposed in this way.

Proof We prove the statement for $l = 2$, and the general statement follows by induction on l that proceeds much along the same lines. First, note that the definition of the strain intersection graph implies the objective function decomposition $\sum_j w_j u_j = \sum_{j \in c_1} w_j u_j + \sum_{j \in c_2} w_j u_j$, where we write $j \in c_1$ if simple strain j can be used to cover some complex strain in c_1, and similarly for $j \in c_2$. Indeed, if there were a simple strain j that could be used to cover a complex strain $i_1 \in c_1$ as well as $i_2 \in c_2$, there would be an edge between i_1 and i_2, contradicting the definition of c_1 and c_2 as disjoint components. By the same reasoning, the constraints involving $j \in c_1$ are disjoint from those involving $j \in c_2$. Finally, for the negative term in the MDL-inspired objective function we also have

the decomposition $\sum_{i,j} c_{ij} \log_2(n_j) = \sum_{i,j \in c_1} c_{ij} \log_2(n_j) + \sum_{i,j \in c_2} c_{ij} \log_2(n_j)$, so both optimization problems indeed separate into two disjoint problems, one for each component. □

Efficiently enumerating optimal solutions

Despite the two-step optimization, the effective number of optimal solutions for a single large connected component sometimes reaches into the thousands. However, these optimal solutions are highly structured. In order to take advantage of this structure, we make use of the following heuristic which allows us to further break down large connected components of the strain intersection graph.

Many complex strains admit only one particular resolution in order for the resulting solution to be optimal. For this reason, we start by generating one optimal solution, and then test, for every complex strain i involved, whether the proposed resolution (defined by one of the two values of j such that $c_{ij} = c_{i(i \setminus j)} = 1$) is required for optimality, by computing the minimum value of the MDL optimization problem with the additional constraint $c_{ij} = 0$. If the resulting problem is either infeasible or has a strictly higher optimum value than the original problem, we can conclude that this particular resolution is required for optimality.

After identifying the subset of the complex strains with a particular resolution required for optimality, we can remove the nodes corresponding to them from the strain intersection graph, which will typically further subdivide the connected component they are part of into smaller ones. Each of these smaller components can also be optimized separately with the constraint that the simple strains j required for resolving the removed nodes are now available at no cost (i.e. $w_j = 0$) to avoid double-charging, yet still provide the bonus if they are used (i.e. $\log_2(n_j)$ for every i such that $c_{ij} = 1$).

In our experiments we use this approach on connected components of size at least $\delta = 6$. In smaller components, we enumerate the optimal solutions by repeatedly adding the constraint $\sum_{j \in S_k} u_j \leq K - 1$ after the kth iteration, where S_k is the set of all simple strains not previously seen in the data that are used to resolve the complex strains at the kth iteration, and K is the minimum possible number of such strains obtained in the first stage of the optimization. We additionally implement a special analysis for components of size 1, since the full set of optimal solutions can be easily found directly in this case.

Simulating datasets

This section describes the procedure we follow to generate our simulated datasets.

Our goal is to produce a number of datasets that share four key features with the dataset of interest: the clustering properties (how well the strains group with one another by similarity); the distribution p_j of the copy numbers at the jth locus; the distribution r_1 of the number of complex loci per strain; and the distribution r_2 of the differences between the copy numbers at the complex loci. We consider only complex strains with content of size 1 or 2, as in our dataset. If $\mathbf{s}$ is a complex strain, then the complex loci are the set $Q = \{1 \leq j \leq L | \ |\mathbf{s}_j| = 2\}$, their number is $q = |Q|$, and the difference between the copy numbers of a complex locus $\mathbf{s}_j = \{a, b\}$ is $|a - b|$.

The process consists of three phases. First, we generate an initial set of patient strains to resemble our target dataset. Second, we evolve this initial dataset over 10 years by a random sequence of mutation and reinfection events, recording the event each time. Third, we randomly select a subset of the patients and create complex strains for a further subset of those. We describe each of these steps in detail.

We start by computing, for $1 \leq j \leq L$, the distribution p_j of copy numbers at the jth locus in our dataset. We also compute the pairwise categorical distances $\mathbf{d_H}$ between all pairs of simple strains (we use categorical distances to avoid biasing the simulation method towards any of the distances we use to classify complex strains), and use these to construct a neighbor-joining tree T [6] whose branch lengths we round up to the next integer. We then randomly generate the strain at the root of the tree, with the jth locus being drawn from the distribution p_j. We recursively generate the other strains in the tree by randomly selecting l out of L loci to mutate along a branch of length l, and assigning them new values based on the probability distributions p_j. Our new dataset is then the set of strains at all the nodes of T. This particular generation method ensures that the strains at the nodes of T have similar clustering properties to those of the original dataset (namely, the families of closely matching strains are likely to be of similar sizes) while the strains themselves are generally very different from those in the original dataset.

The second stage consists of subjecting the dataset to mutation and reinfection events. This differs between the datasets we simulate to look like our initial dataset and those which we simulate to explore the performance of our method under different combinations of mutation and reinfection rates.

To simulate our initial dataset, we perform mutations and reinfections separately to match the overall distributions r_1 and r_2. For the mutations we take the strains at the leaves of the tree T and compute the distribution of the number of complex loci and differences between the complex loci needed to match r_1 and r_2. For each strain s we then select a value q at random from the scaled distribution r_1, pick q loci $j_1, \ldots, j_q$ at random, then pick q values $n_1, \ldots, n_q$ at random from the scaled distribution r_2 and q random directions $d_1, \ldots, d_q \in \{-1, 1\}$. The mutated version of the strain is obtained by changing s_{j_i} to $s_{j_i} + d_i n_i$ for $1 \leq i \leq q$. Any negative values produced become 0 and any values exceeding $t_{\max}$ become $t_{\max}$. For the reinfections we use the strains at the internal nodes of the tree T, pairing them up in such a way as to have the resulting distributions r_1 and r_2 match those of the original dataset as closely as possible. We do so by drawing the appropriate number of pairs for each value of the number of complex loci, choosing among them according to the target probability distribution r_2. Namely, if the pair of strains $\mathbf{s}$ and $\mathbf{s}'$ differs at q distinct loci with differences $\delta_1, \ldots, \delta_q$ respectively, it is picked among all those with q differing loci with a probability proportional to $\Pi_{i=1}^{q} r_2(\delta_i)$. The reason we pick internal nodes rather than leaf nodes as sources of reinfection is to ensure that the underlying simple strains differ from the simple strains in the dataset, which are sampled from the leaf nodes, as otherwise the resolution and classification problems might generate biased results.

On the other hand, to explore the performance of our method under different combinations of mutation and reinfection rates we choose a mutation rate μ per locus per year and a reinfection rate β per person per year, and evolve the dataset for $\tau = 10$ years. For N strains there are a total of $\mu L N \tau$ mutation events and $\beta N \tau$ reinfection events. At the start of the simulation every patient has two identical strains, but all mutations affect the second strain to allow for accumulation (while not necessarily biologically realistic, this choice ensures that the two strains in each patient look as different as possible from one another). For each event we draw a random patient to be affected. For mutation events we draw the locus to be mutated in the affected patient's strain randomly, then mutate it up or down by 1 with equal probability, unless it's either 0 or $t_{\max}$, in which case we use the only admissible direction of mutation. For reinfection events we draw a random source patient and let one of her strains replace one of the affected patient's strains, marking the affected patient as reinfected. We consider a range of mutation rates computed experimentally by Ragheb et al [7]. We note that, although the true value of this mutation rate is controversial, this range includes both estimates by Reyes and Tanaka [8, 9] and Supply et al [10, 11]. We take the medium mutation rate to be $\mu_{med} = 0.0012$ per locus per year, half the mean estimate of Ragheb et al [7], and define the high and low mutation rates $\mu_{hi} = 0.012$ and $\mu_{lo} = 0.00012$. Following Verver et al [12] we take the medium reinfection rate to be $\beta_{med} = 0.04$ per

person per year, twice the rate in treated TB patients, and define the high and low reinfection rates $\beta_{hi} = 0.12$ and $\beta_{lo} = 0.012$.

The third and last stage consists of selecting a set of patients at random from the final dataset. Out of the selected patients, we further select the desired number of clonally heterogeneous strains and the desired number of mixed infection strains (those coming from reinfected patients); the remaining patients only contribute one strain each, chosen at random among the two strains they harbor. This becomes the simulated dataset.

We evaluate the closeness of the simulated dataset to the original dataset by computing the total variation distance [13] between their respective distributions r_1 and r_2. The sum of these total variation distances gives a measure of the closeness, with values between 0.05 and 0.1 for the dataset made to resemble the original dataset and between 0.25 and 0.5 for the exploratory datasets. We find that in order to attain these reasonable distances, it is helpful to exclude any patients with more than $q = 12$ complex loci or a difference of more than $\delta = 7$ between copy numbers at a complex locus (both being the maximum values observed in the actual dataset) in stage 3. For each dataset we use a starting set in stage 1 which showed good performance in a preliminary test, then perform 100 iterations of stage 2 and for each resulting dataset, 100 iterations of stage 3. We then select the top 100 closest simulated datasets out of the resulting samples and use these for our simulations. Each of them has $N = 415$ strains, of which 42 are clonally heterogeneous and 41 are mixed infections (for the exploratory datasets we choose $N = 355$ strains per dataset, with 36 clonally heterogeneous and 35 mixed infections in each).

Selection of the best method for ClassTR

Based on the results of **Table 2**, we choose to use the weighted linear model for classifying the strains once a pool of simple strains has been created. This is primarily due to the fact that $\mathbf{d_L^w}$ has the best discriminatory power, making ties for the closest distance the most unlikely - indeed, we observe very few ties when using it, so most probabilities end up being 0 or 1. Furthermore, this is also the metric that yields the best average performance on the simulated datasets.

References

1. Jiang T, Ravikumar B. Minimal NFA Problems are Hard. SIAM J Comput. 1993;6(22):11171141.

2. Rissanen J. A Universal Prior for Integers and Estimation by Minimum Description Length. Annals of Statistics. 1983;11(2):416431.

3. Yang Y, Bickel D. Minimum Description Length and Empirical Bayes Methods of Identifying SNPs Associated with Disease. No. 74 in Collection of Biostatistics Research Archive; 2010. p. 1-23.

4. Hraber P, Korber B, Wolinsky S, Erlich H, Trachtenberg E, Kepler T. HLA and HIV Infection Progression: Application of the Minimum Description Length Principle to Statistical Genetics. In: Maglaveras N, Chouvarda I, Koutkias V, Brause R, editors. Biological and Medical Data Analysis. vol. 4345 of Lecture Notes in Computer Science. Springer Berlin Heidelberg; 2006. p. 1-12.

5. Chaitankar V, Zhang C, Ghosh P, Gong P, Perkins E, Deng Y. Predictive Minimum Description Length Principle Approach to Inferring Gene Regulatory Networks. In: Arabnia HR, Tran QN, editors. Software Tools and Algorithms for Biological Systems. vol. 696 of Advances in Experimental Medicine and Biology. Springer New York; 2011. p. 37-43.

6. Saitou N, Nei M. The neighbor-joining method: a new method for reconstructing phylogenetic trees. Molecular Biology and Evolution. 1987;4(4):406-425.

7. Ragheb M, Ford C, Chase M, Lin P, Flynn J, Fortune S. The mutation rate of mycobacterial repetitive unit loci in strains of M. tuberculosis from cynomolgus macaque infection. BMC Genomics. 2013;1(14):145.

8. Reyes J, Tanaka M. Mutation rates of spoligotypes and variable numbers of tandem repeat loci in Mycobacterium tuberculosis. Infection, Genetics and Evolution. 2010;10:1046-1051.

9. Reyes J, Tanaka M. Mutation rate of VNTR loci in Mycobacterium tuberculosis: Response to Supply et al. Infection, Genetics and Evolution. 2011;11:1189-1190.

10. Supply P, Niemann S, Wirth T. On the mutation rates of spoligotypes and variable numbers of tandem repeat loci of Mycobacterium tuberculosis. Infection, Genetics and Evolution. 2011;11:251-252.

11. Supply P, Niemann S, Wirth T. On the mutation rates of spoligotypes and variable numbers of tandem repeat loci of Mycobacterium tuberculosis: Continued-When tuning matters. Infection, Genetics and Evolution. 2011;11:1191.

12. Verver S, Warren R, Beyers N, Richardson M, van der Spuy G, Borgdorff M, et al. Rate of reinfection tuberculosis after successful treatment is higher than rate of new tuberculosis. American Journal of Respiratory and Critical Care in Medicine. 2005;171:1430-1435.

13. Levin D, Peres Y, Wilmer E. Markov Chains and Mixing Times. 1st ed. American Mathematical Society; 2008.
